# Global research progress of endothelial cells and ALI/ARDS: a bibliometric analysis

**DOI:** 10.3389/fphys.2024.1326392

**Published:** 2024-05-07

**Authors:** Tong Zhou, Kunlan Long, Jun Chen, Lijia Zhi, Xiujuan Zhou, Peiyang Gao

**Affiliations:** ^1^ Department of Critical Care Medicine, Hospital of Chengdu University of Traditional Chinese Medicine, Chengdu, China; ^2^ School of Clinical Medicine, Chengdu University of Traditional Chinese Medicine, Chengdu, China

**Keywords:** bibliometric analysis, acute lung injury, endothelial cells, hotspots, acute respiratory distress syndrome

## Abstract

**Background:**

Acute lung injury (ALI) and acute respiratory distress syndrome (ARDS) are severe respiratory conditions with complex pathogenesis, in which endothelial cells (ECs) play a key role. Despite numerous studies on ALI/ARDS and ECs, a bibliometric analysis focusing on the field is lacking. This study aims to fill this gap by employing bibliometric techniques, offering an overarching perspective on the current research landscape, major contributors, and emerging trends within the field of ALI/ARDS and ECs.

**Methods:**

Leveraging the Web of Science Core Collection (WoSCC) database, we conducted a comprehensive search for literature relevant to ALI/ARDS and ECs. Utilizing Python, VOSviewer, and CiteSpace, we performed a bibliometric analysis on the corpus of publications within this field.

**Results:**

This study analyzed 972 articles from 978 research institutions across 40 countries or regions, with a total of 5,277 authors contributing. These papers have been published in 323 different journals, spanning 62 distinct research areas. The first articles in this field were published in 2011, and there has been a general upward trend in annual publications since. The United States, Germany, and China are the principal contributors, with Joe G. N. Garcia from the University of Arizona identified as the leading authority in this field. *American Journal of Physiology-Lung Cellular and Molecular Physiology* has the highest publication count, while *Frontiers in Immunology* has been increasingly focusing on this field in recent years. “Cell Biology” stands as the most prolific research area within the field. Finally, this study identifies endothelial glycocalyx, oxidative stress, pyroptosis, TLRs, NF-κB, and NLRP3 as key terms representing research hotspots and emerging frontiers in this field.

**Conclusion:**

This bibliometric analysis provides a comprehensive overview of the research landscape surrounding ALI/ARDS and ECs. It reveals an increasing academic focus on ALI/ARDS and ECs, particularly in the United States, Germany, and China. Our analysis also identifies several emerging trends and research hotspots, such as endothelial glycocalyx, oxidative stress, and pyroptosis, indicating directions for future research. The findings can guide scholars, clinicians, and policymakers in targeting research gaps and setting priorities to advance the field.

## 1 Introduction

Acute lung injury (ALI) and acute respiratory distress syndrome (ARDS) were first proposed by Ashbaugh and colleagues in 1967 (Ashbaugh et al., 1967). In 1994, the American-European Consensus Conference (AECC) characterized ALI as the rapid onset of respiratory failure, with a PaO_2_/FiO_2_ ratio ≤300 mmHg, irrespective of the level of positive end-expiratory pressure, bilateral infiltrates on chest X-ray, and pulmonary artery wedge pressure ≤18 mmHg without evidence of left atrial hypertension. ARDS shares the same criteria but requires a lower PaO_2_/FiO_2_ ratio of <200 mmHg (Bernard et al., 1994; Raghavendran and Napolitano, 2011). Although ALI/ARDS manifests as a “syndrome” induced by various injuries and diseases, they share similarities in pathophysiology, clinical manifestations, and specific targets for pharmacological intervention. Hence, ALI and ARDS are often studied collectively (Hu et al., 2022). Current therapeutic strategies primarily focus on symptom alleviation and supportive care, such as mechanical ventilation and fluid restriction, while also addressing the underlying injury or disease (Gorman et al., 2022). Nonetheless, even with aggressive treatment, patients still face a high in-hospital mortality rate ranging from 30%–43% (Bellani et al., 2016; Moss et al., 2019), and the majority of survivors experience long-term physical, psychological, and/or cognitive impairments. Therefore, the pressing task at hand is to develop effective therapies that can inhibit the pulmonary inflammatory cascade, thereby reducing the mortality and long-term morbidity induced by ALI/ARDS.

The pathogenesis of ALI/ARDS is complex, involving the activation and imbalance of multiple interrelated injury response pathways, along with extensive pulmonary and systemic inflammatory reactions, edema, and coagulation abnormalities (Bos and Ware, 2022). Within this intricate landscape of physiological and pathological phenomena, the dysfunction and injury of pulmonary microvascular endothelial cells (ECs) hold a central position (Bos and Ware, 2022). ECs serve as a crucial barrier between the circulatory system and pulmonary parenchyma and airways. Impairment in ECs primarily manifests as an expansion of intercellular gaps and upregulation of adhesion molecules like P-selectin and E-selectin, as well as other mediators of endothelial injury such as vascular endothelial growth factor-2 (VEGF-2) (Mulligan et al., 1992; Newman et al., 1993; Parikh et al., 2006). These alterations are triggered by a variety of factors, including circulating pathogens or their metabolic products, endogenous disease-related molecules, pro-inflammatory cytokines, and free hemoglobin (Hough et al., 2019). Interestingly, ECs are enveloped by a delicate structure known as the endothelial glycocalyx (EG). When subjected to various stimuli, this structure can degrade, resulting in the unveiling of adhesive molecules that consequently facilitate edema formation (Schmidt et al., 2012). Additionally, anticoagulant molecules on the ECs surface, such as antithrombin and endothelial protein C receptor, may also be shed due to injury, while pro-coagulant factors become upregulated, further contributing to microvascular thrombus formation (Livingstone et al., 2021). Therefore, ECs play a pivotal role in both the pathogenesis and clinical outcomes of ALI/ARDS. To effectively prevent ALI/ARDS, restore pulmonary microvascular barrier function, mitigate pulmonary inflammatory responses, improve patient prognosis, and reduce the risk of mortality, a deep understanding of the mechanisms of ECs injury in ALI/ARDS holds significant clinical and research implications.

Bibliometrics serves as a tool with the advantage of quantitatively analyzing research trends and hotspots across multiple disciplines and industries, including but not limited to management, sociology, economics, medicine, environmental engineering, and agriculture (Sun et al., 2022a). This methodology is adept at systematically mining the scientific knowledge embedded in a wealth of unstructured data in established fields, revealing emerging research trends and collaboration patterns, as well as organizing the knowledge structure within specific fields (Donthu et al., 2021). Consequently, bibliometrics not only enables scholars to gain a comprehensive overview of existing research but also identifies knowledge gaps, proposes innovative research directions, and elucidates potential contributions to the field. Despite the plethora of publications on ALI/ARDS and ECs, there has yet to be a study that systematically explores their interrelation through bibliometric methods. The present study aims to fill this void by employing bibliometric techniques to quantitatively analyze the current state of research on ECs in ALI/ARDS, including key contributors, collaboration patterns, high-impact journals, disciplinary knowledge structure, and research trends, thereby providing a scientific basis for future research directions and principal issues in this field.

## 2 Materials and methods

### 2.1 Search strategy

To obtain a comprehensive view of research related to ALI/ARDS and ECs, we conducted an exhaustive literature search in the Web of Science Core Collection (WoSCC) database. Given the rapid updates of the database content, the search activity was completed within 28 July 2023, to ensure data timeliness. This study spans a publication period from 1985 to 2023. The search query employed was as follows: TS=(“acute respiratory distress syndrome*" OR “acute lung injury”) AND TS=(“vascular endothelium” OR “vascular endothelial cell*" OR “endothelium” OR “endothelial cell*"). To ensure research quality and comparability, document types were restricted to “Article” and “Review Article”, and only publications in English were included. The retrieved articles were subsequently added to the “Marked List” in the Web of Science personal account for further analysis and screening.

### 2.2 Data acquisition

**FIGURE 1:**
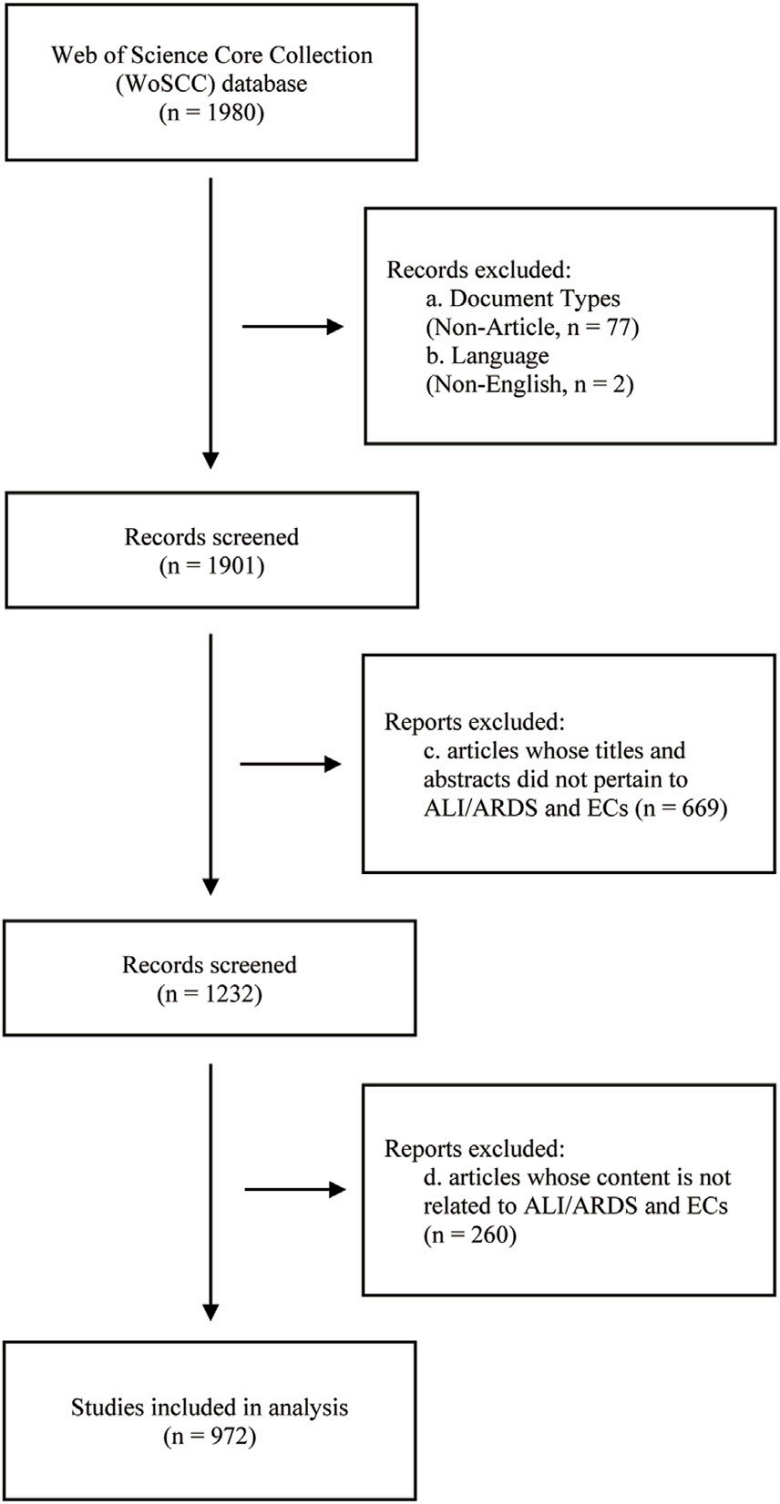
Flowchart of literature search selection.

To ensure the accuracy and reliability of literature screening, two authors independently reviewed the titles and abstracts of all retrieved articles. In the preliminary screening, articles whose titles and abstracts did not pertain to ALI/ARDS and ECs were excluded. Subsequently, articles that mentioned ALI/ARDS and ECs in their titles or abstracts but were not relevant to the research objectives or content were also eliminated. After comparing the screening results from both parties, any discrepancies were resolved through mutual discussion or consultation with a third-party expert. This screening process was completed on 21 August 2023, with detailed steps outlined in Figure 1.

Following this, all eligible articles were exported from the Web of Science personal account’s “Marked List” as a “Plain Text File”, which included the “Full Record and Cited References”. Additionally, the built-in “Analyze Results” and “Citation Report” features of the Web of Science database were also exported. Details are as follows:

a. In the “Plain Text File”, various pieces of information about the articles were located in different field tags. For instance, the PT field tag indicated the beginning of an article record, while the ER field tag signified its end. The author’s full name was stored in the AF field tag, keywords in the DE field tag, complete correspondence addresses of all authors (including affiliated countries and institutions) in the C1 field tag, citation count in the TC field tag, publication year in the PY field tag, and the research field in the WC field tag. This data was used for bibliometric analyses using Python, VOSviewer, and CiteSpace.
b. The inherent “Analyze Results” functionality of Web of Science offered a breakdown of publications by year, nation, institution, author, and journal, facilitating the verification and validation of outputs from Python, VOSviewer, and CiteSpace.
c. The database’s “Citation Report” was leveraged to aggregate the annual citation count, acting as an index of the research’s significance.

The methods aim to offer a comprehensive, rigorous, and accurate bibliometric analysis, laying a robust foundation for further research.

### 2.3 Data preprocessing

Prior to embarking on a detailed bibliometric examination, we meticulously processed the raw data utilizing Python. The detailed steps we adopted are elucidated below:

a. Country Classification: Notably, in international statistics, some regions or countries might be listed separately, even though they are geographically or politically part of the same entity. To address this issue, we implemented a merging operation. For example, Wales, Scotland, England, and Northern Ireland were consolidated under “United Kingdom".
b. Author Keyword Standardization: To avoid information loss due to the presence of keyword synonyms, we performed deduplication and synonym replacement for author keywords. Specifically, “ALI” and “acute lung injury” were standardized as “acute lung injury (ALI)". The complete synonym table is provided in Supplement 1.
c. Author Name Verification: To eliminate confusion arising from authors with the same names, we conducted thorough identity verification following prior research methodologies (Sun et al., 2022b). Initially, we revisited the Web of Science database and re-searched for publications associated with specific author names under the original search criteria. Subsequently, for those authors with disputed identities, we employed multiple verification methods, including clear differentiation among authors with identical but non-identical names, authors with matching initials but different full names, and authors whose names had slight spelling variations but were indeed the same individual. In addition to utilizing ORCID information for identity validation, we also consulted official institutional websites and encyclopedias among other reliable sources.

Based on these verification outcomes, we carried out necessary partitioning or merging operations to ensure the accuracy and reliability of the bibliometric analysis.

### 2.4 Data analysis

This study leveraged the Python to extract multiple key attributes from the literature, such as publication year, authors, institutions, countries, research areas, journals, references, and author keywords, followed by comprehensive statistical analysis. These analyses included calculations of total publications, total citation frequency, and h-index across various dimensions like years, authors, institutions, countries, and research areas. Notably, the h-index, initially used to quantify the academic impact of an individual researcher, has now been expanded to assess the collective academic caliber of entities like academic teams, institutions, and nations (Hirsch, 2005; Braun et al., 2006; Van Raan, 2006; Csajbók et al., 2007). To visually represent the data, we also produced bubble charts illustrating the annual publication trends within various research areas, journals, and author keywords. In these charts, the diameter of the bubble symbolizes the most prominent research areas, journals, or author keywords for that year, while numbers within the bubbles indicate the corresponding publication quantity and thematic frequency (Sun et al., 2022b). All Python codes employed for data preprocessing and analysis are publicly available on GitHub (https://github.com/changecool/WOS_bibliometric-analysis).

To delve into the structural nuances of collaborative networks and thematic clusters, we employed VOSviewer 1.6.19 software for visualization analysis. In the generated network diagrams, individual nodes can represent different countries, institutions, authors, or keywords. The size of the nodes reflects the number of publications attributed to them, while distinct colors denote different clusters or years. The connecting lines between nodes reveal relationships of collaboration or citation.

Additionally, we also utilized CiteSpace 6.2.R3 software for burst analysis on references and author keywords to identify key references and research themes that have emerged prominently within specific time periods. In the burst analysis results chart, blue lines represent time intervals, whereas red lines indicate the starting and ending years covered by references or author keywords exhibiting burst phenomena.

## 3 Results

### 3.1 Publication summary

**FIGURE 2:**
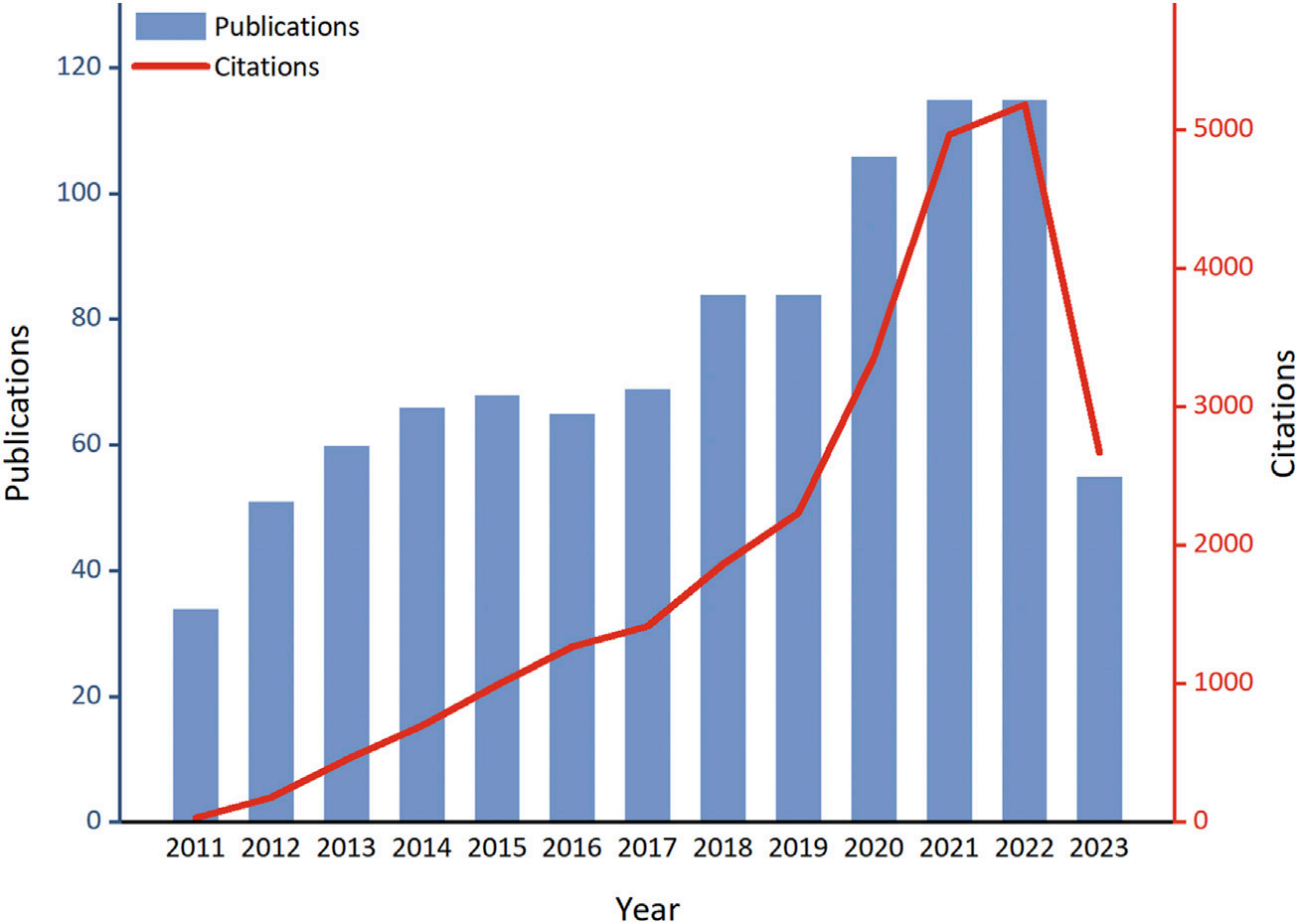
Trends in the growth of publications and the number of citations in ALI/ARDS and ECs.

Through a search in the WoSCC database, a total of 1,901 papers related to ALI/ARDS and ECs were retrieved, with no duplicate publications found. After evaluation by two independent authors, 929 papers unrelated to the research theme were excluded, leaving 972 papers for effective inclusion in the analysis. Among these, 851 were original research articles (accounting for 87.55%), and 121 were review articles (comprising 12.45%). Examined from a temporal perspective, both the publication volume and citation frequency in this research field have displayed a marked growth trend from 2011 to 2023 (Figure 2). Specifically, only 34 related articles were published in 2011, while by 2021 and 2022, the annual publication volumes reached 115 papers each, underscoring a significant elevation in research activity within this field. The first half of 2023 has already seen 55 relevant publications; if this trend persists, the annual publication volume is anticipated to remain consistent with the previous 2 years. On the other hand, citation counts have also risen significantly, further confirming the escalating impact and recognition of research in this field. In 2011, the citation count for related papers was merely 36, but this figure soared dramatically starting in 2019, reaching 5,182 by 2022—an increase of over 143 times compared to 2011. Notably, despite only half of 2023 having elapsed, papers in this field have already been cited 2,675 times, signaling that their impact is set to continue its ascent.

### 3.2 Contribution of countries/regions

Regarding research on ALI/ARDS and ECs, we reviewed 972 academic papers from 40 countries or regions. Table 1 provides a detailed breakdown of key metrics such as publication counts, citation counts, and h-index for the ten most productive countries or regions. Comprehensive analysis of the literature data from 2011 to 2023 clarified the contributions of various nations within this research field. In terms of publication volume, China leads with 437 articles, closely followed by the United States with 426 articles; the influence of these two nations in this field is particularly significant. Other countries that excelled in total paper count (TP) include Germany (60), Japan (45), and the United Kingdom (36). Regarding the impact of papers, as gauged by citation counts, the United States (13,836) significantly outperformed China (7,532). Furthermore, we calculated the average citation per paper (ACPP), a metric derived by dividing the total citation count (TC) by the total paper count (TP). This relative measure may more accurately reflect the attention garnered by the articles. In this respect, Germany led with an ACPP value of 54.37, followed by the United Kingdom (44.61), France (43.25), and Greece (40.36). It is noteworthy that the United States ranked first in h-index with 59. Additionally, we incorporated metrics related to international collaboration, including the number of collaborating countries (nCC) and the share of multi-country collaborative papers (SMCP). In this aspect, the United States excelled, collaborating with 25 countries and producing 142 multi-country collaborative articles. Taking all these parameters into account, the data reveal that the United States, Germany, and the United Kingdom excel not only in publication count but also in citation frequency, h-index, and international collaboration. In contrast, although China possesses a clear advantage in article quantity, its papers receive comparatively less attention.

**TABLE 1:** Contribution of the top 20 countries/regions in ALI/ARDS and ECs.

Rank	Country	TP	TC	h-index	ACPP	nCC	MP	SMCP
1	China	437	7,532	42	17.24	12	66	15.1
2	United States	426	13,836	59	32.48	25	142	33.33
3	Germany	60	3,262	29	54.37	19	47	78.33
4	Japan	45	1,668	21	37.07	6	16	35.56
5	United Kingdom	36	1,606	20	44.61	17	27	75
6	Brazil	25	526	14	21.04	8	19	76
7	Canada	24	943	17	39.29	11	15	62.5
8	France	16	692	12	43.25	13	9	56.25
9	Greece	14	565	11	40.36	10	3	21.43
10	Italy	14	322	8	23	9	12	85.71

TP, total papers; TC, total citations; ACPP, average citations per publication; nCC, number of cooperative countries; SMCP, share of multinational cooperation publications; and MP, multinational publications.

**FIGURE 3:**
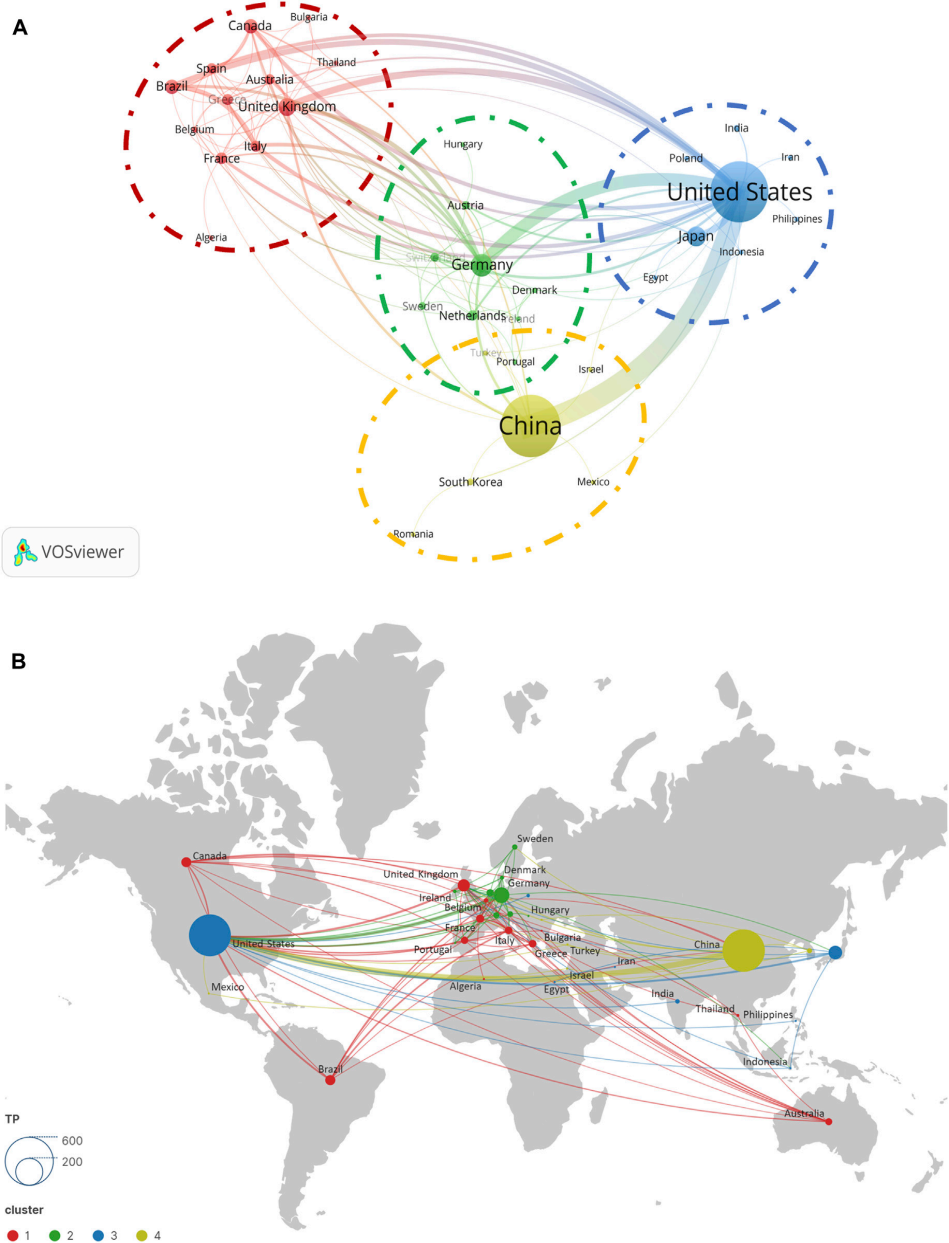
Cooperation map of countries/regions **(A)** and geographical distribution **(B)** in ALI/ARDS and ECs.

In the process of co-author analysis using VOSviewer software, we constructed a network map (Figure 3A) representing collaborations between countries/regions. During the optimization of the network map, nodes that did not participate in international collaboration were excluded. After this filtration, 35 countries were retained in the map as subjects of study. According to VOSviewer’s algorithm, these countries were divided into four distinct clusters. It is noteworthy that the United Kingdom, Germany, the United States, and China spearhead their respective clusters, which aligns with our previous analysis concerning national influence. Examining the collaboration patterns within each cluster, we observed:

a. Intensive collaborations exist among the United Kingdom, Brazil, Canada, France, Greece, Italy, Spain, Australia, Belgium, Thailand, Algeria, and Bulgaria.
b. Germany demonstrates close collaborations with the Netherlands, Switzerland, Austria, Sweden, Denmark, Ireland, Hungary, and Portugal.
c. In the cluster led by the United States, frequent collaborations are observed with Japan, India, Poland, Egypt, Indonesia, Iran, and the Philippines.
d. China maintains strong collaborative ties with South Korea, Israel, Mexico, Romania, and Turkey.

Lastly, from the geographic distribution of national collaborations (Figure 3B), Europe, North America, and Asia emerge as the principal hotspots for cooperation, with Europe’s collaboration network being particularly dense.

### 3.3 Contribution of institutions

In the field of ALI/ARDS and ECs research, we identified contributions from 978 different research institutions. Notably, among the top 20 institutions listed in Table 2, China and the United States are represented by 10 and 8 institutions respectively, with the remaining two hailing from Brazil and Canada. Using the number of publications as a metric, Univ Illinois stands out, also securing the highest rankings in both TC and h-index. When considering ACPP, Univ Pittsburgh in the United States and Southeast Univ in China are tied for first place with a score of 53.07, followed by Univ Toronto (39.11) and Univ Illinois (38.39). Although Chinese institutions are most numerous in the top 20 list, American institutions outperform in total publications, total citations, and ACPP.

**TABLE 2:** Contribution of the top 20 institutions in ALI/ARDS and ECs.

Rank	Institution	TP	TC	h-index	ACPP	TPR(%)	Country
1	Univ Illinois	77	2,956	33	38.39	7.92	United States
2	Shanghai Jiao Tong Univ	41	887	17	21.63	4.22	China
3	Univ Arizona	38	777	16	20.45	3.91	United States
4	Univ Chicago	34	1,308	23	38.47	3.5	United States
5	Fudan Univ	25	803	14	32.12	2.57	China
6	Brown Univ	22	590	16	26.82	2.26	United States
7	Nanjing Med Univ	22	306	10	13.91	2.26	China
8	Univ Maryland	21	368	12	17.52	2.16	United States
9	Univ Penn	20	662	16	33.1	2.06	United States
10	Wuhan Univ	20	230	10	11.5	2.06	China
11	China Med Univ	19	369	9	19.42	1.95	China
12	Wenzhou Med Univ	19	276	10	14.53	1.95	China
13	Univ Toronto	18	704	14	39.11	1.85	Canada
14	Anhui Med Univ	16	89	5	5.56	1.65	China
15	Chongqing Med Univ	15	270	8	18	1.54	China
16	Univ Pittsburgh	14	743	11	53.07	1.44	United States
17	Southeast Univ	14	743	9	53.07	1.44	China
18	Univ Sao Paulo	14	311	7	22.21	1.44	Brazil
19	Univ Rochester	14	266	10	19	1.44	United States
20	Fourth Mil Med Univ	14	177	9	12.64	1.44	China

TP, total papers; TC, total citations; ACPP, average citations per publication; and TPR, the percentage of articles of institutions in total publications.

**FIGURE 4:**
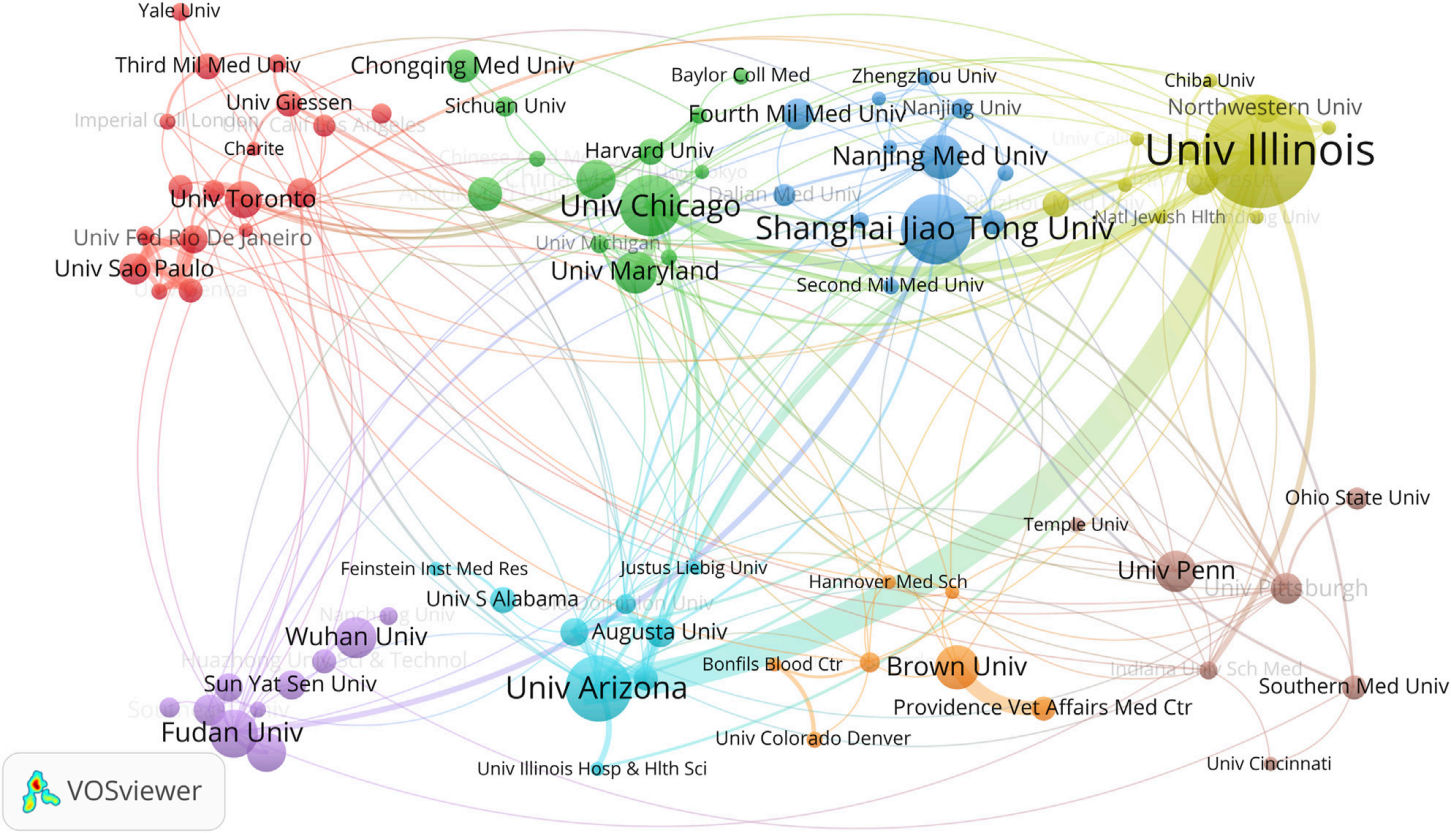
Cooperation map of institutions in ALI/ARDS and ECs.

Furthermore, we employed VOSviewer software for co-authorship analysis and constructed an institutional collaboration network map (Figure 4). We set a minimum publication count of 5 as the participation threshold, and 88 institutions met this criterion. After removing unconnected nodes, the network map ultimately comprised 87 institutions. Among them, Univ Toronto, Univ Chicago, Shanghai Jiao Tong Univ, Univ Illinois, Fudan Univ, Univ Arizona, Brown Univ and Univ Penn serve as the focal points of their respective clusters, all ranking within the top 20 in terms of productivity. In terms of collaboration frequency, Univ Illinois and Univ Arizona collaborate most frequently, followed by Univ Chicago and Univ Illinois. This observation aligns with Univ Illinois’ leading position in TC, h-index, and publication count.

### 3.4 Contribution of authors

In the field of ALI/ARDS and ECs research, statistical analysis of 5,277 authors across 972 publications yielded substantive data on top experts in the field. Specifically, 4,295 authors published only a single article, while 124 authors have published five or more articles. Even more scarce are authors with ten or more publications, amounting to only 28 individuals. The top 20 prolific authors and their affiliated institutions are summarized in Table 3. Collectively, they account for 324 publications, constituting 33.33% of all research articles in this field. Notably, Garcia, Joe G. N. emerges as the most prolific author in terms of publication count and also leads in TC and h-index, thereby establishing himself as the most authoritative figure in the ALI/ARDS and ECs field. Of these top 20 authors, 17 are based in the United States, one in Germany, and two in China. This data further underscores the concentration of ALI/ARDS and ECs research in specific countries. Three academic institutions—Univ Arizona (United States), Univ Maryland (United States), and Univ Illinois (Germany)—each have three prolific authors, corroborating our earlier findings on institutional contributions.

**TABLE 3:** Contribution of the top 20 authors in ALI/ARDS and ECs.

Rank	Author	TP	TC	h-index	ACPP	Institution	Country
1	Garcia, Joe G. N	41	1,400	23	34.15	Univ Arizona	United States
2	Birukova, Anna A	32	733	17	22.91	Univ Maryland	United States
3	Dudek, Steven M	25	736	13	29.44	Univ Illinois Hosp and Hlth Sci Syst	United States
4	Birukov, Konstantin G	25	628	15	25.12	Univ Maryland	United States
5	Wang, Ting	17	410	13	24.12	Univ Arizona	United States
6	Black, Stephen M	17	383	12	22.53	Florida Int Univ	United States
7	Jacobson, Jeffrey R	16	529	11	33.06	Univ Illinois	United States
8	Sammani, Saad	14	499	12	35.64	Univ Arizona	United States
9	Verin, Alexander D	13	369	11	28.38	Augusta Univ	United States
10	Lu, Qing	13	341	10	26.23	Univ Arizona Hlth Sci	United States
11	Tian, Yufeng	13	309	11	23.77	Univ Chicago	United States
12	Malik, Asrar B	12	826	11	68.83	Univ Illinois	United States
13	Letsiou, Eleftheria	12	400	9	33.33	Humboldt Univ	Germany
14	Mehta, Dolly	11	631	9	57.36	Univ Illinois	United States
15	Fazal, Fabeha	11	229	9	20.82	Univ Rochester	United States
16	Camp, Sara M	11	202	8	18.36	Univ Arizona	United States
17	Karki, Pratap	11	154	7	14	Univ Maryland Hosp	United States
18	Catravas, John D	10	319	8	31.9	Old Dominion Univ	United States
19	Yang, Yi	10	310	7	31	Southeast Univ	China
20	Qiu, Haibo	10	308	6	30.8	Southeast Univ	China

TP, total papers; TC, total citations; and ACPP, average citations per publication.

**FIGURE 5:**
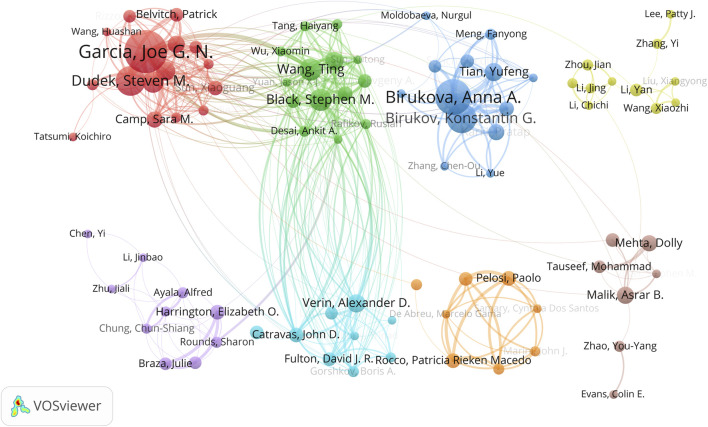
Cooperation map of authors in ALI/ARDS and ECs.

A detailed author collaboration network was generated using VOSviewer software (Figure 5). With a minimum paper count threshold set at five, 124 authors met this criterion. After removing unconnected nodes from the network, a total of 88 authors were included. This collaboration network was further divided into eight distinct author clusters. Particularly noteworthy are four closely collaborating clusters: the red cluster led by Garcia, Joe G. N., the green cluster led by Black, Stephen M. and Wang, Ting, the blue cluster led by Birukova, Anna A., and the light blue cluster led by Verin, Alexander D. These leading authors all rank within the top 10 in terms of productivity, which is consistent with the data presented in Table 3.

### 3.5 Contribution of journals

Between 2011 and 2023, a total of 972 research articles related to ALI/ARDS and ECs were published across 323 different journals. Specific ranking data is presented in Table 4. The most active among them is the *American Journal of Physiology-Lung Cellular and Molecular Physiology* with 65 articles, followed by the *American Journal of Respiratory Cell and Molecular Biology* (40 articles, accounting for 4.12%), *PLOS ONE* (29 articles, 2.98%), *Shock* (24 articles, 2.47%), and *International Immunopharmacology* (20 articles, 2.06%). In terms of total citations over the past decade, the *American Journal of Physiology-Lung Cellular and Molecular Physiology* was cited 1,886 times, followed by *PLOS ONE* (1,782 times) and the *American Journal of Respiratory Cell and Molecular Biology* (1,259 times). Interestingly, journals with fewer articles, such as the *American Journal of Respiratory and Critical Care Medicine*, achieved a high ACPP of 96 with just eight articles. This may be related to the journal’s high Impact Factor (IF) and its broad scope of content (Garfield, 2006).

**TABLE 4:** Contribution of the top 20 journals in ALI/ARDS and ECs.

Rank	Journal	TP	TC	ACPP	IF	TPR(%)
1	American Journal of Physiology-Lung Cellular and Molecular Physiology	65	1886	29.02	4.9	6.69
2	American Journal of Respiratory Cell and Molecular Biology	40	1,259	31.48	6.4	4.12
3	PLOS ONE	29	1782	61.45	3.7	2.98
4	Shock	24	432	18	3.1	2.47
5	International Immunopharmacology	20	262	13.1	5.6	2.06
6	Scientific Reports	18	479	26.61	4.6	1.85
7	Frontiers in Immunology	17	230	13.53	7.3	1.75
8	Respiratory Research	16	442	27.62	5.8	1.65
9	FASEB Journal	16	252	15.75	4.8	1.65
10	Pulmonary Circulation	14	346	24.71	2.6	1.44
11	International Journal of Molecular Sciences	14	277	19.79	5.6	1.44
12	Cellular Physiology and Biochemistry	12	413	34.42	0	1.23
13	Cells	11	106	9.64	6	1.13
14	Frontiers in Physiology	11	70	6.36	4	1.13
15	Journal of Immunology	10	497	49.7	4.4	1.03
16	Microvascular Research	10	292	29.2	3.1	1.03
17	Biochemical and Biophysical Research Communications	10	119	11.9	3.1	1.03
18	Frontiers in Pharmacology	10	64	6.4	5.6	1.03
19	American Journal of Respiratory and Critical Care Medicine	8	768	96	24.7	0.82
20	European Respiratory Journal	8	656	82	24.3	0.82

TP, total papers; TC, total citations; ACPP, average citations per publication; and TPR, the percentage of articles of institutions in total publications.

Further analysis reveals that most articles pertaining to ALI/ARDS and ECs have an ACPP significantly higher than their respective IF, reflecting the relatively large number of scholars in the field and higher citation rates for these articles. As for the IF, except for *Cellular Physiology and Biochemistry*, which was not included in the latest 2023 Journal Citation Reports, the highest value was for the *American Journal of Respiratory and Critical Care Medicine* (24.7), followed by the *European Respiratory Journal* (24.3), *Frontiers in Immunology* (7.3), *American Journal of Respiratory Cell and Molecular Biology* (6.4), and *Cells* (6).

**FIGURE 6:**
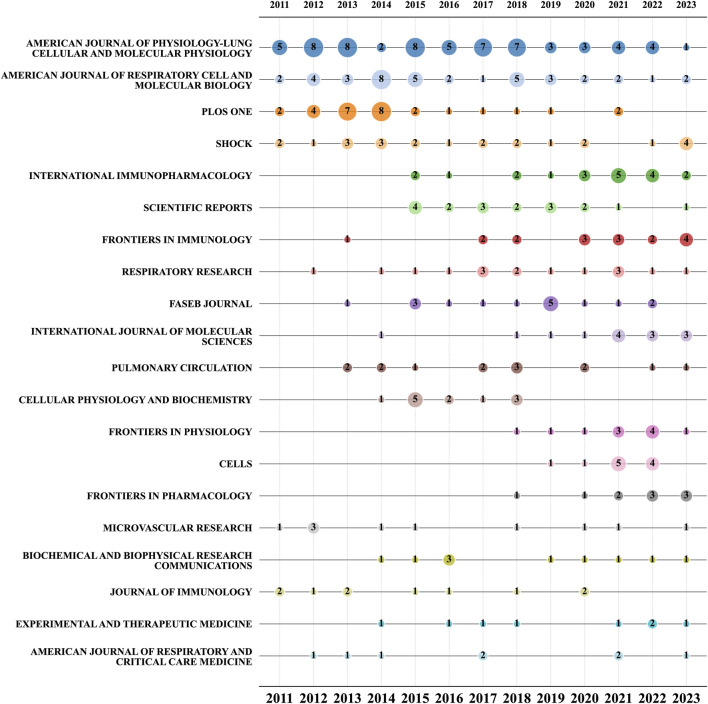
Bubble chart of the top 20 journals by year.

Figure 6 presents a bubble chart of the top 20 journals related to ALI/ARDS and ECs. The chart reveals that from 2011 to 2022, the *American Journal of Physiology-Lung Cellular and Molecular Physiology* has consistently been the most prolific journal in this research field. Notably, although the *American Journal of Respiratory Cell and Molecular Biology* and *PLOS ONE* rank among the top three in overall publication volume, they have shown a general decline since 2016. Conversely, between 2020 and 2023, journals such as *International Immunopharmacology*, *Frontiers in Immunology*, *International Journal of Molecular Sciences*, *Frontiers in Physiology*, and Cells have experienced a noticeable increase in publication volume. Specifically, *Frontiers in Immunology* has already published four relevant studies in the first half of 2023.

### 3.6 Contribution of research areas

In the realm of ALI/ARDS and ECs research, a total of 62 distinct research areas are encompassed. The top 20 areas with the highest publication volume are detailed in Supplementary Table S1. Among these, “Cell Biology” leads with 199 articles, followed by “Respiratory System” and “Biochemistry & Molecular Biology”. Notably, these three areas account for 56.06% of all published articles. However, they do not exhibit a significant advantage in terms of ACPP. This suggests that although these areas contribute significantly to publication volume, the corresponding high citation counts are not conspicuous. Conversely, the top three areas in terms of ACPP— “Infectious Diseases” (61.71), “Materials Science, Multidisciplinary” (56.5), and “Nanoscience & Nanotechnology” (51.4)—have relatively fewer published articles, with 7, 4, and 5 papers respectively, but garner more substantial attention and citations.

**FIGURE 7:**
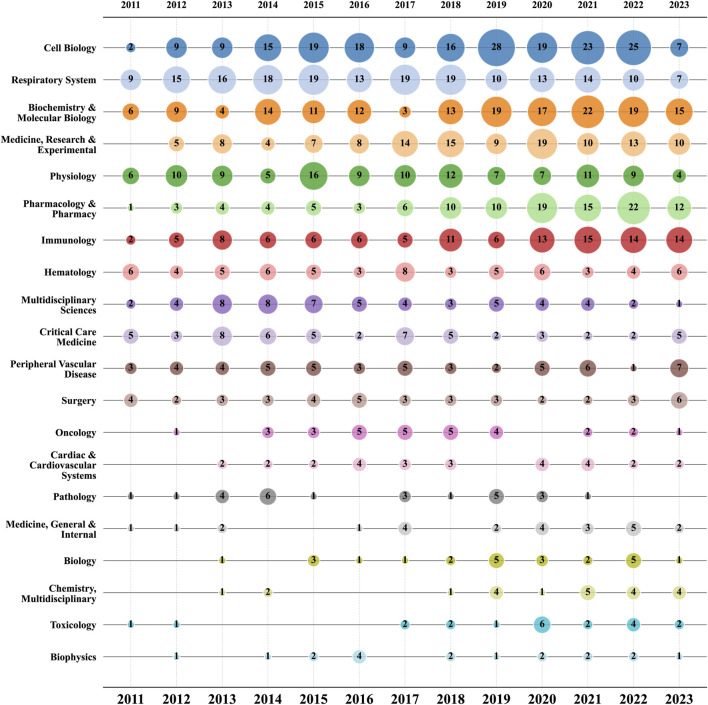
Bubble chart of the top 20 research areas by year.

Figure 7 further presents a bubble chart of the top 20 research areas by publication volume in the fields of ALI/ARDS and ECs. The data reveal that “Cell Biology”, “Respiratory System”, and “Biochemistry & Molecular Biology” have consistently been the most prolific areas in terms of publications from 2011 to 2022, underscoring their enduring significance in the overall research landscape. Worth noting is that, beginning in 2018, certain research areas—particularly “Pharmacology & Pharmacy” and “Immunology"—have experienced a noticeable surge in publication volume from 2020 to 2022.

### 3.7 Analysis of ESI highly cited papers and citation bursts references

In the body of literature on ALI/ARDS and ECs, a total of 972 articles were analysed. Of these, 16 articles were categorized as Essential Science Indicators (ESI) highly cited papers, published within the last decade and ranking in the top 1% of global citations in the past 2 months, as shown in Supplementary Table S2. Specifically, 10 of these articles were published prior to 2020, while 6 were published between 2021 and 2023. Among these six recently published ESI high-impact articles, four are primarily review articles focusing on “COVID-19/SARS-CoV-2/Coronavirus”, delving into the pathogenesis and therapeutic approaches related to ALI/ARDS in the context of COVID-19. One article, authored by Biancatelli, Ruben M. L. Colunga et al. (Biancatelli et al., 2021), demonstrates that the S1 subunit of the SARS-CoV-2 spike protein can induce acute lung injury similar to COVID-19 in K18-hACE2 transgenic mice, leading to endothelial barrier dysfunction. Another article, by Silva, Johnatas Dutra et al. (Silva et al., 2021), reveals that in an ARDS environment, mitochondrial function in alveolar epithelial cells and endothelial cells is compromised. Mesenchymal stromal cell-derived extracellular vesicles can at least partially restore mitochondrial function via mitochondrial transfer, thereby improving alveolar-capillary barrier performance.

**FIGURE 8:**
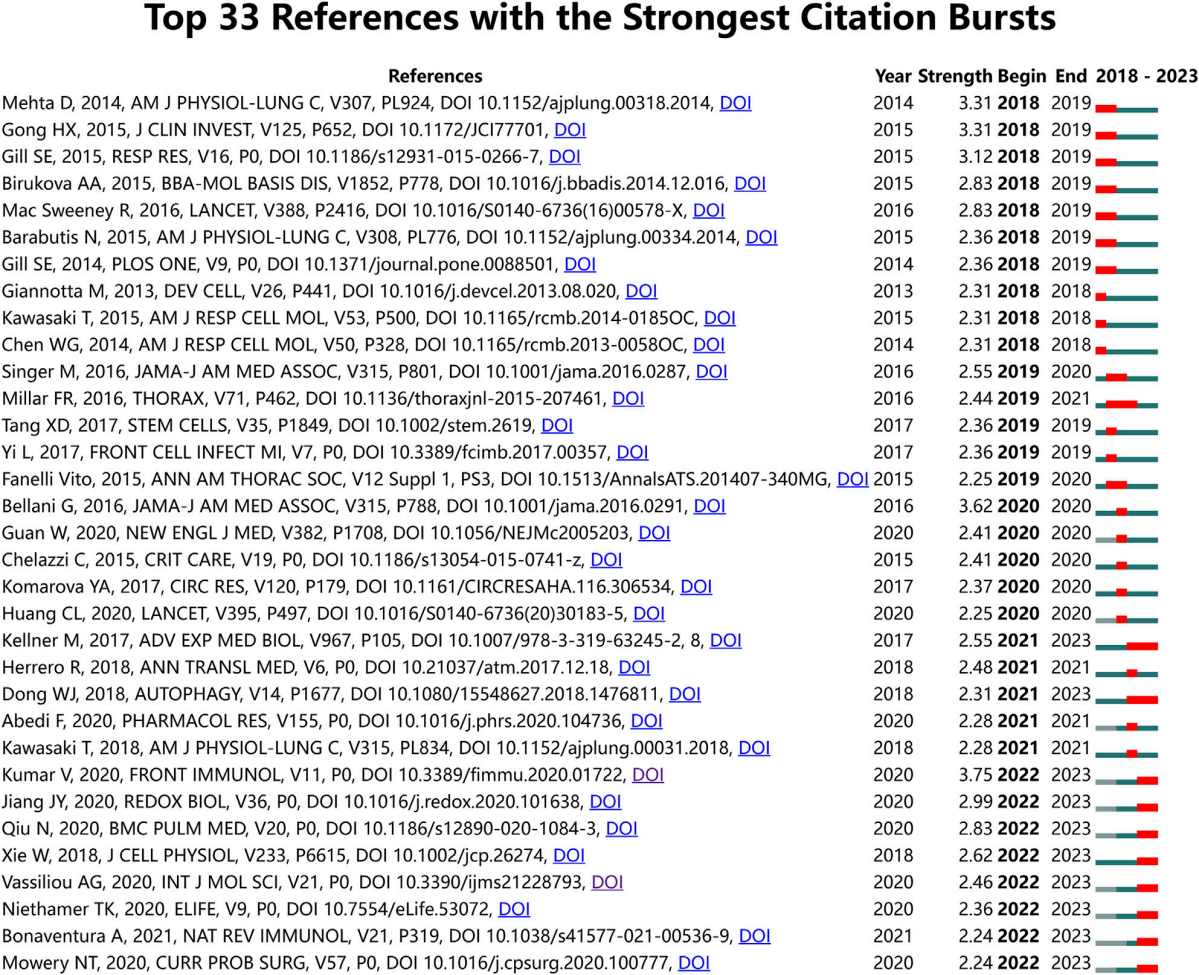
All references with the strongest citation bursts in ALI/ARDS and ECs.

For an overarching view of literature citations, we utilized CiteSpace software to perform Burstness analysis on the references of the 972 articles in the ALI/ARDS and ECs filed. The time span was set from 2018 to 2023, with a Minimum Duration set at 1 year. The analysis revealed that there are 33 references with a significant upward trend in citations; of these, 10 exhibited burst citations as of 2023, and four references had a burst strength greater than 2.50 (Figure 8). The reference (Kumar, 2020) with the highest burst strength (3.75) primarily explores the immunological mechanisms inducing ALI in pneumonia and sepsis but does not discuss endothelial cells. Another reference by Jiang JY et al. (Jiang et al., 2020) (with a burst strength of 2.99) investigates the role of NOX4 in causing pulmonary endothelial barrier dysfunction in ALI/ARDS. Qiu N et al. (Qiu et al., 2020) (with a burst strength of 2.83) reports that long non-coding RNA TUG1 ameliorates endothelial cell inflammation and apoptosis in sepsis-induced ALI by targeting miR-34b-5p and GAB1. Xie W et al. (Xie et al., 2018) (with a burst strength of 2.62) found that in LPS-induced ALI, miR-34b-5p mitigates endothelial cell inflammation and apoptosis by targeting PGRN. These studies collectively suggest that NOX4, lncRNA TUG1, and miR-34b-5p not only have pivotal roles in the pathogenesis of ALI/ARDS but may also serve as effective therapeutic targets for this condition.

### 3.8 Analysis of author keywords

Author keywords often provide abundant information and have thus become a focal point of broad interest. Analyzing the evolving trends in author keywords can track research frontiers and predict hotspots and trends in the field (Sun et al., 2022b). Initially, we consolidated and standardized 2082 author keywords, filtering down to 1,419 unique keywords. Notably, 218 articles did not provide author keywords and were consequently excluded from the statistical analysis. Of these 1,419 keywords, 1,079 (76.04%) appeared only once, 305 (21.49%) appeared 2–10 times, 23 (1.62%) appeared 11–20 times, five (0.35%) appeared 21–50 times, and seven (0.49%) appeared between 51 and 550 times. The top 30 most frequently occurring keywords cumulatively appeared 1,670 times, accounting for 42.85% of the total occurrences (3,897).

**FIGURE 9:**
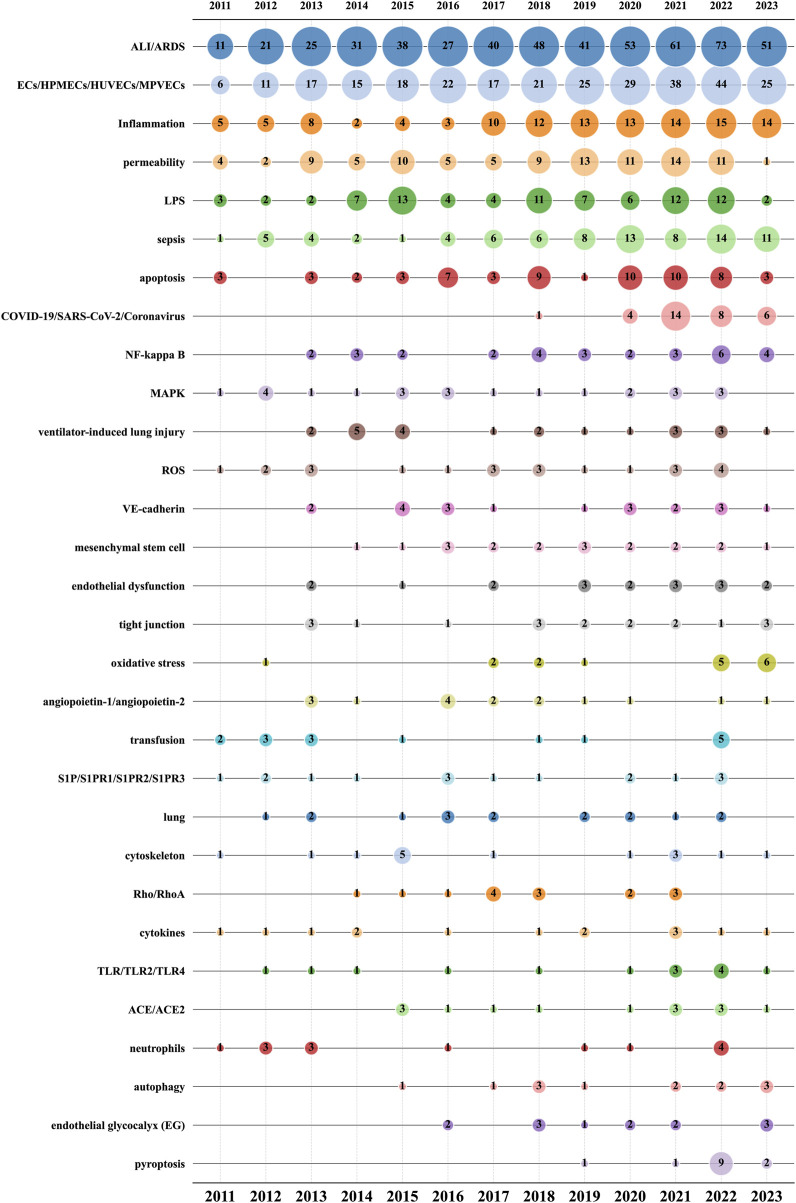
Bubble chart of the top 30 author keywords by year.

Figure 9 further refines the dynamic characteristics of author keyword usage, showing data across three dimensions: publication year, author keyword, and corresponding article count. “ALI/ARDS” (520) and “ECs/HPMECs/HUVECs/MPVECs” (288), the two core keywords in this research field, occupy the top positions and have been increasing annually, reflecting the yearly publication trend in this field.

Among the remaining 28 keywords:

a. Seven focus on diseases and symptoms: “inflammation” (118), “sepsis” (83), “LPS” (85), “COVID-19/SARS-CoV-2/Coronavirus” (33), “ventilator-induced lung injury” (23), “transfusion” (16), “lung” (16).
b. Eleven pertain to cellular biological processes or mechanisms: “apoptosis” (62), “permeability” (99), “mesenchymal stem cell” (19), “endothelial dysfunction” (18), “tight junction” (18), “oxidative stress” (17), “cytoskeleton” (15), “neutrophils” (14), “endothelial glycocalyx” (13), “autophagy” (13), “pyroptosis” (13).
c. Ten are related to signaling pathways and molecular routes: “NF-kappa B” (31), “MAPK” (24), “ROS” (23), “VE-cadherin” (20), “angiopoietin-1/angiopoietin-2” (16), “S1P/S1PR1/S1PR2/S1PR3” (16), “Rho/RhoA” (15), “cytokines” (14), “TLR/TLR2/TLR4” (14), “ACE/ACE2” (14).

Amid the COVID-19 pandemic in 2020, research related to “COVID-19/SARS-CoV-2/Coronavirus” surged, peaking in 2021. Notably, in cellular biology, the frequency of research on three keywords: “endothelial glycocalyx”, “oxidative stress”, and “pyroptosis” has noticeably increased in recent years. “Endothelial glycocalyx” has had 13 related publications since its introduction into the field in 2016. “Oxidative stress” had six articles in the first half of 2023, while “pyroptosis” saw an explosive increase in 2022 with nine articles. In the realm of signaling pathways and molecular routes, “NF-kappa B″ reached a new publication high in 2022 with a total of six articles. Additionally, starting from 2021, “TLR/TLR2/TLR4” has also exhibited a significant research growth trend. These shifts mirror the evolution in research focus and methodology, and may also indicate new directions and possibilities for treating ALI/ARDS and similar conditions.

**FIGURE 10:**
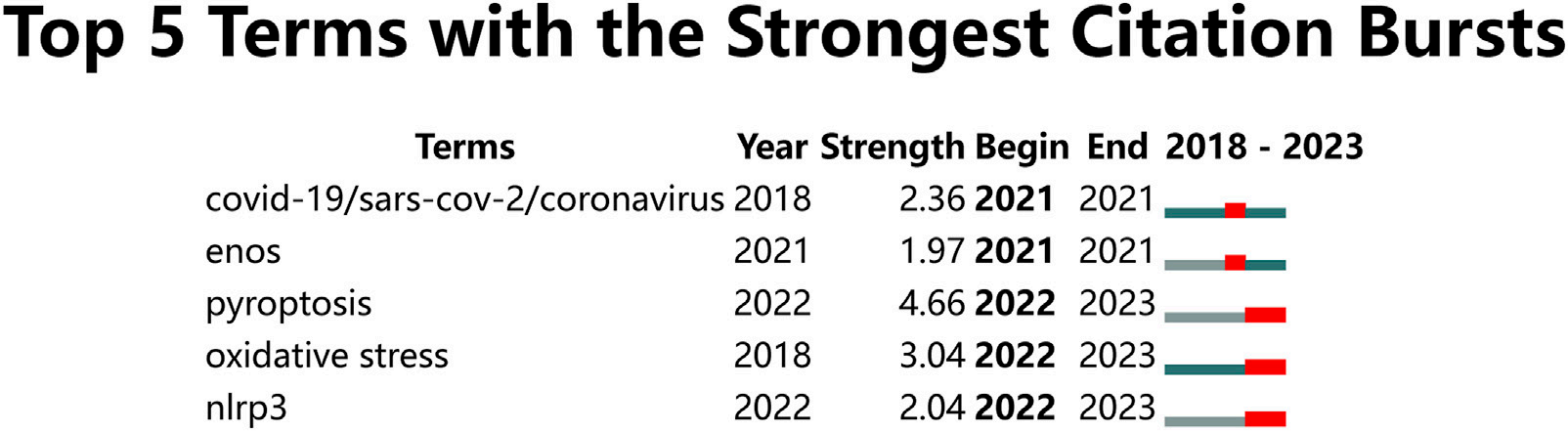
All author keywords with the strongest citation bursts in ALI/ARDS and ECs.

**FIGURE 11:**
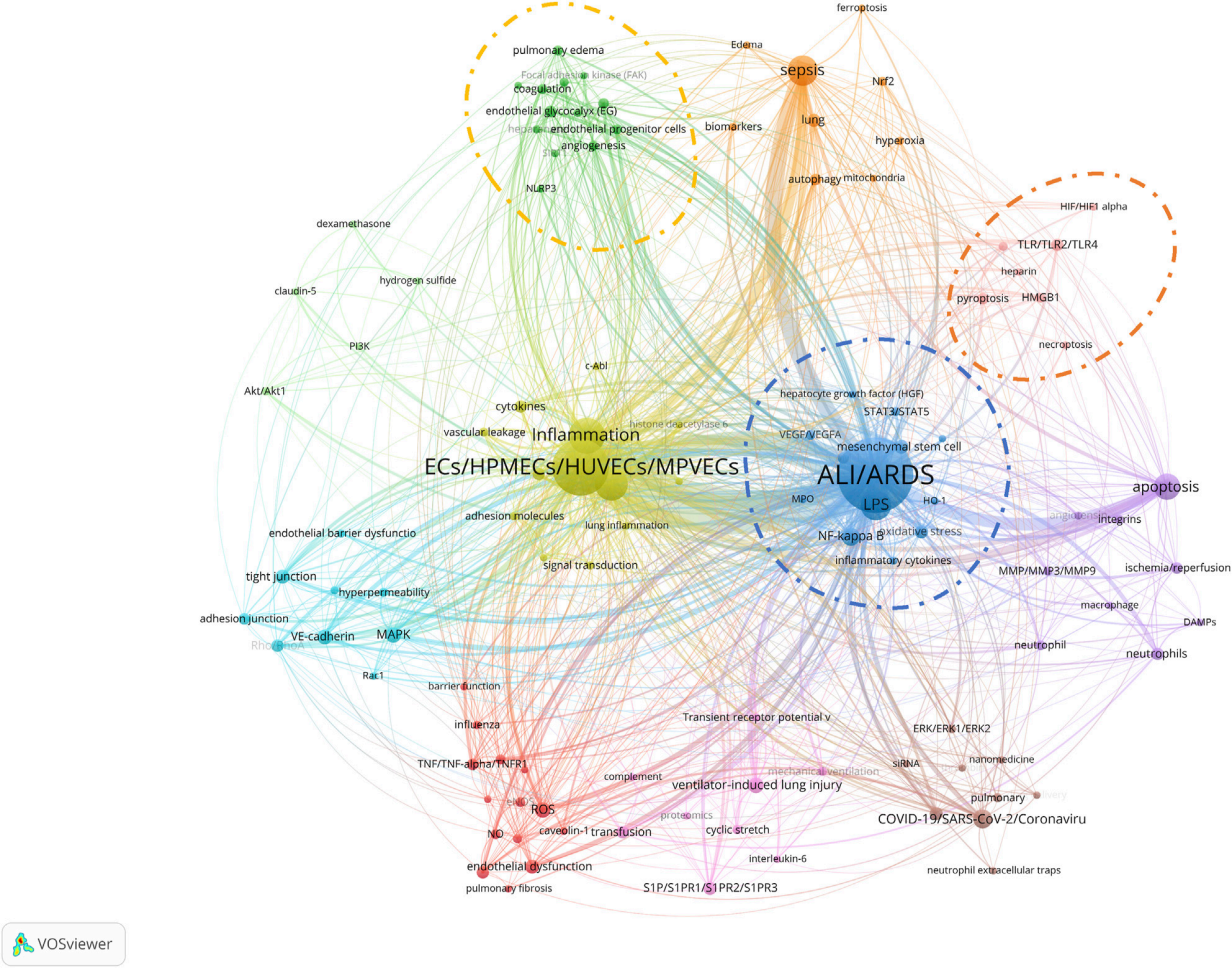
Co-occurrence network map of author keywords in ALI/ARDS and ECs.

To precisely identify keywords that may have a significant impact on ALI/ARDS and ECs research in recent years, we employed CiteSpace software to perform a citation burst analysis on the author keywords of 972 articles. The analysis timeframe was set from 2018 to 2023, with the “Minimum Duration” parameter set to 1 year. The results revealed that five author keywords demonstrated significant trends in citation growth. Notably, as of 2023, three keywords—"pyroptosis”, “oxidative stress”, and “NLRP3″—stood out prominently (Figure 10). Specifically, the publication counts for “pyroptosis” and “oxidative stress” have noticeably risen in the past 2 years, corroborating the data in the bubble chart. Although research on “NLRP3” has a total publication count of only six articles and did not make it into the top 30, all these articles were published between 2022 and 2023. This finding indicates that “pyroptosis”, “oxidative stress”, and “NLRP3” are undeniably among the hotspot topics in the ALI/ARDS and ECs research field over the last 2 years. Lastly, through co-occurrence network analysis using VOSviewer, we further explored the interrelationships among the keywords. In the co-occurrence analysis within VOSviewer software, an author keyword co-occurrence network map was constructed (Figure 11), setting the minimum frequency for each author keyword at five publications. Among the 1,419 author keywords, 110 met this threshold, identifying 11 co-occurring clusters of author keywords. Three of these clusters encompassed the research hotspot keywords in the ALI/ARDS and ECs fields, as identified in the bubble chart and citation burst analysis:

a. Within the cluster encircled by a blue dashed line, the author keywords included: “ALI/ARDS”, “LPS”, “NF-kappa B″, “mesenchymal stem cell”, “oxidative stress”, “ICAM-1″, “VEGF/VEGFA”, “STAT3/STAT5”, “inflammatory cytokines”, “hepatocyte growth factor (HGF)", “HO-1″, “MPO”, and “severe acute pancreatitis".
b. Within the cluster encircled by a red dashed line, the author keywords included: “TLR/TLR2/TLR4”, “pyroptosis”, “HMGB1”, “caspase-1/caspase-3/caspase-11″, “heparin”, “necroptosis”, and “HIF/HIF1 alpha".
c. Within the cluster encircled by a yellow dashed line, the author keywords included: “endothelial glycocalyx”, “miRNA”, “coagulation”, “pulmonary edema”, “angiogenesis”, “endothelial progenitor cells”, “extracellular vesicles (EVs)", “exosomes”, “heparanase”, “Focal adhesion kinase (FAK)", “NLRP3”, “SIRT1”, and “thrombosis".

## 4 Discussion

### 4.1 General information

From 2011 to 2023, academic research in the fields of ALI/ARDS and ECs has demonstrated a significant upward trend, especially in terms of citation counts, indicating increasing recognition and influence in this field. Geographically, although China and the United States publish a nearly equal number of papers, the United States wields greater influence in terms of citations and h-index. Meanwhile, Germany and the United Kingdom excel in ACPP, suggesting higher research quality from these nations. International collaborations are primarily concentrated in Europe, North America, and Asia, with Europe being the most intensive collaborator. Institutionally, Univ Illinois in the U.S. excels in the number of published papers, citations, and h-index, solidifying its authority in this field. In contrast, despite China’s predominance in the number of publications, its performance in citations and ACPP is relatively weaker. These findings not only elucidate the relative influence and contributions of various stakeholders in the ALI/ARDS and ECs fields but also offer valuable insights for future research directions and resource allocation.

In the realm of ALI/ARDS and ECs, prolific authors are predominantly from the United States, followed by Germany and China. Moreover, most high-output authors are affiliated with institutions that perform well in institutional contribution analyses, such as Univ Arizona, Univ Maryland, and Univ Illinois, further emphasizing the role of institutions in generating high-quality research. The research leadership in this field is concentrated in the hands of a few prolific authors, notably Garcia, Joe G. N., from Univ Arizona, who is considered the most authoritative expert based on his outstanding performance in both paper count and h-index. From the perspective of journals, the *American Journal of Physiology-Lung Cellular and Molecular Physiology* holds a leading position in this field. Emerging journals like *Frontiers in Immunology* have shown significant growth in recent years, indicating their potential importance in the future. From the angle of research areas, “Cell Biology”, “Respiratory System”, and “Biochemistry and Molecular Biology” dominate in terms of publication volume but lack a significant advantage in ACPP, implying these areas may not necessarily attract broad academic attention. In contrast, more niche research areas like “Infectious Diseases” and “Materials Science, Multidisciplinary”, though fewer in publication count, show higher ACPP, signifying greater academic focus.

Viewed from the lens of ESI highly cited literature, research from 2021 to 2023 mainly concentrates on reviews related to “COVID-19/SARS-CoV-2/Coronavirus.” This trend is likely influenced by the ESI evaluation mechanism, which typically prioritizes papers cited in the top 1% globally over the past decade. Particularly under the impact of global events like the COVID-19 pandemic, research topics closely related to the crisis naturally gain heightened attention. In these publications, ECs play a critical role in the pathogenesis of ALI/ARDS, especially as the subject of studies on endothelial barrier dysfunction. This explains the frequent appearance of endothelial cells in ALI/ARDS research related to “COVID-19/SARS-CoV-2/Coronavirus.” Compared to COVID-19-related research focusing mainly on ESI highly cited literature, citation bursts up to 2023 pay more attention to ALI/ARDS and ECs themselves. As an evaluative tool, citation bursts more accurately capture long-term research trends or gradually emerging fields. According to citation burst analyses, current high-impact literature mainly focuses on immune mechanisms, oxidative stress, lncRNAs, and miRNAs. These research directions not only occupy the frontier of ALI/ARDS pathogenesis studies but also foreshadow their potential pivotal roles in future research and treatment modalities.

### 4.2 Research hotspots and trends

Based on a composite analysis of bubble charts for author keywords and citation burst analyses, research related to author keywords such as “endothelial glycocalyx”, “oxidative stress”, “pyroptosis”, “NF-kappa B″, “TLR/TLR2/TLR4”, and “NLRP3” has seen significant growth in recent years in the fields of ALI/ARDS and ECs. This not only confirms the rising academic interest in these themes but also further suggests their likely emergence as focal points and cutting-edge directions for future research.

#### 4.2.1 Endothelial glycocalyx

EG is a gel-like structure that coats the surface of endothelial cells (ECs), serving as a negatively charged macromolecular barrier, extracellular molecular receptor, and mechanical stress sensor. It is implicated in various physiological processes, including vascular permeability regulation, coagulation, inflammatory responses, and signal transduction (Foote et al., 2022). Primarily composed of glycosaminoglycans such as heparan sulfate (HS), chondroitin sulfate (CS), and hyaluronic acid (HA), these macromolecules are anchored by transmembrane proteins like syndecans and glypicans. Notably, heparin sulfate proteoglycans (HSPGs), formed by HS and transmembrane proteins, constitute a significant component of the EG, playing a crucial role in its structure and integrity (Foote et al., 2022). EG degradation is considered a key mechanism leading to vascular leakage and alveolar injury in ALI/ARDS, principally facilitated by glycocalyx-degrading enzymes like heparinase (HPA) and metalloproteinases (MMPs). This degradation compromises the integrity of the EG, initiating a cascade of pathological changes, such as the loss of the macromolecular charge barrier and dysfunction of vascular tone regulatory mechanisms (Milusev et al., 2022). Recent studies have also discovered that EG degradation further impacts the expression of endothelial junctional proteins, thereby compromising vascular integrity (Mensah et al., 2017). These collective pathological changes result in increased pulmonary microvascular permeability, culminating in diffuse alveolar damage. Thus, the EG is garnering increasing attention in ALI/ARDS and ECs research and is likely to become a significant research direction.

Currently, research on EG in the ALI/ARDS field focuses mainly on two avenues: Firstly, it is broadly considered a potential therapeutic target. Several drug candidates have been evaluated for efficacy and safety against EG in animal models, such as Tanshinone IIA (El-Moslemany et al., 2022), SERP 30 polysaccharide (Feng et al., 2022), Crocin (Zhang et al., 2020), and PCTR1(35). These candidates primarily target the expression of EG degradation products, like syndecan-1, or enzymes like HPA. Clinical studies have shown that high-dose intravenous vitamin C can significantly reduce ARDS-associated syndecan-1 levels, further validating EG as an effective therapeutic target (Qiao et al., 2022). Secondly, EG-related biomarkers are being examined in clinical studies to determine their reliability as indicators for ARDS diagnosis or prognosis. A prospective cohort study focusing on pediatric ARDS related to sepsis revealed a clear clinical outcome correlation with plasma EG degradation product patterns, particularly HS and syndecan-1 (Sallee et al., 2023). This study found higher levels of HS and syndecan-1 in the plasma of affected children, independently associated with reduced ventilator-free days at 28 days, thereby substantiating the potential clinical value of EG-related biomarkers. In these major research directions, EG status is typically assessed indirectly through its degradation products, rather than direct observation, mainly due to the immaturity of direct observation techniques and lack of extensive validation (Foote et al., 2022). Thus, overcoming this technical challenge could further promote the application and development of EG in ALI/ARDS research.

Integrating author keyword co-occurrence network analysis with existing literature, we identify significant overlaps in the ALI/ARDS and ECs research fields with EG-related author keywords like “SIRT1” and “exosomes”. Activation of sirtuin 1 (SIRT1) protein in endothelial cells can inhibit EG degradation by downregulating HPA expression, thereby ameliorating ALI in mouse models (Li et al., 2020; Wang et al., 2021). As for the intersection with exosomes, HPA can increase exosome release via the syndecan-syntenin-Alix pathway, playing a vital role in the onset and progression of ARDS (Feng et al., 2023). Although other keywords like “endothelial progenitor cells”, “extracellular vesicles”, and “miRNA” also belong to the same author keyword cluster as EG, there has yet to be research reported in the ALI/ARDS field. However, in other disease models, exosomes or extracellular vesicles from various cell sources, including endothelial progenitor cells, carrying miRNAs have been proven to mitigate EG degradation (Zhang Y. et al., 2021; Li Z. et al., 2023). This presents an unexplored research direction for ALI/ARDS. Additionally, other keywords in the same cluster, like “Focal Adhesion Kinase (FAK)" and “NLRP3”, also lack research pertaining to EG, opening up new possibilities for future studies.

#### 4.2.2 Oxidative stress

Oxidative stress represents a pathological condition characterized by an overproduction of reactive oxygen species (ROS) and reactive nitrogen species (RNS) that exceeds the neutralizing or scavenging capacity of intracellular or tissue-based antioxidant systems, such as superoxide dismutase (SOD), catalase, and reduced glutathione (GSH) (Joffre and Hellman, 2021). This imbalance leads to excessive generation of ROS and RNS, thereby activating ECs and inducing the release of various pro-inflammatory cytokines (e.g., TNF-α, IL-1, IL-6) and chemokines (e.g., MCP-1). Under conditions of oxidative stress, expression of cell adhesion molecules, such as intercellular adhesion molecule-1 (ICAM-1) and vascular cell adhesion molecule-1 (VCAM-1), is upregulated. This amplifies leukocyte-endothelial interactions, further exacerbating vascular permeability and leading to pathological phenomena like pulmonary edema (Scioli et al., 2020). More alarmingly, excessive ROS can trigger multiple endothelial cell death mechanisms, including pyroptosis, parthanatos, and ferroptosis, thereby aggravating lung tissue damage (Zheng et al., 2022). Notably, this damage is bidirectional: activated or damaged ECs in turn amplify the inflammatory response, leading to further production of ROS and RNS, thus creating a vicious cycle. In summary, oxidative stress plays a pivotal role in the pathogenesis of ALI/ARDS, not only as a major contributor to early endothelial dysfunction but also as an integral aspect of ALI/ARDS pathophysiology.

Given the widespread consideration of oxidative stress as a key component in the pathophysiology of ALI/ARDS, related biomarkers such as ROS are frequently employed as evaluation metrics in preclinical drug efficacy trials. While most existing research primarily focuses on the therapeutic efficacy of novel preclinical drugs using oxidative stress as an evaluation metric, these studies nonetheless exhibit distinct innovative approaches. Specifically, they delve into the diverse molecular mechanisms and signaling pathways that modulate oxidative stress, and through this avenue, several novel regulatory molecules and pathways have been identified that can ameliorate ECs damage by inhibiting oxidative stress, thereby providing new avenues for improving the clinical status of ALI/ARDS. For instance, Cai et al. (Cai et al., 2022) revealed in a study on burn-induced acute lung injury (ALI) that activation of the Notch pathway in pulmonary microvascular endothelial cells (PMVECs) significantly reduced ROS production and cell apoptosis by downregulating NOX4 expression. This study elucidated the critical role of the Notch-NOX4 signaling pathway in regulating ROS generation and cell apoptosis in PMVECs, thus offering a new therapeutic target for burn-induced ALI. In addition to this, activation of pathways such as JAK2/STAT3 (Hu M. et al., 2022), downregulation of TLR4 expression (Jiang et al., 2021), inhibition of the MAPK/NF-κB pathway (Ju et al., 2022), and activation of the Nrf2/HO-1 signaling pathway (Yang et al., 2016) have all been reported to possess the potential to inhibit ROS production and ameliorate ECs function. Although these reports still lack comprehensive and in-depth validation, they offer a diverse array of therapeutic targets for improving ECs functional impairment in ALI/ARDS via molecular-level modulation of oxidative stress.

In the co-occurrence network analysis, most of the keywords that cluster with “oxidative stress”, such as “NF-kappa B”, “ICAM-1”, “STAT3/STAT5”, “inflammatory cytokines”, and “HO-1” have been previously discussed, demonstrating a significant intersection between ALI/ARDS and ECs research and “oxidative stress.” However, a few keywords like “hepatocyte growth factor”, “MPO”, and “VEGF/VEGFA” are still underrepresented in the literature within this research field concerning oxidative stress. These keywords could signify areas warranting further exploration, especially regarding the interaction mechanisms between oxidative stress and ALI/ARDS.

#### 4.2.3 Pyroptosis

Pyroptosis is a form of programmed cell death distinct from apoptosis and necrosis (Ju et al., 2022). This process can be activated by various types of inflammasomes, such as NLRP3, AIM2, and NLRC4. During activation, pro-caspase-1—an inactive precursor of caspase-1—is converted to its active form. Subsequently, active caspase-1 initiates the cleavage of gasdermin D (GSDMD), leading to the formation of membrane pores. Besides caspase-1, other caspases like caspase-4, caspase-5 (in humans), and caspase-11 (in mice) may also participate in pyroptosis (Yang et al., 2016). Importantly, the activation of caspase-1 concurrently triggers the activation of inflammatory cytokines IL-1β and IL-18, intensifying its proinflammatory characteristics. Pyroptosis has been demonstrated to play a pivotal role in endotoxin-induced lung injury, suggesting that inhibiting endothelial cell pyroptosis could be a critical strategy for treating ALI/ARDS (Cheng et al., 2017).

In recent years, as the understanding of the pathogenesis of ALI/ARDS deepens, endothelial cell pyroptosis has emerged as a focal point of research. The subject has been widely discussed from various angles, including molecular mechanisms, therapeutic strategies, and etiology. Firstly, from an inflammatory perspective, the classic activation pathways of endothelial cell pyroptosis, particularly involving NLRP3 inflammasomes and Caspase-1 components, hold significant implications in the pathogenesis of ALI/ARDS (Liu B. et al., 2021). Specifically, in LPS-induced ALI models, the drug metformin can significantly reduce endothelial cell pyroptosis by inhibiting NLRP3 activity (Zhang et al., 2022). Moreover, recent studies have revealed that high-mobility group box 1 (HMGB1) can also activate Caspase-1 in hemorrhagic shock-induced ALI, thus amplifying LPS-triggered endothelial cell pyroptosis (Yang et al., 2016). Secondly, in terms of pharmacological interventions, beyond metformin, small molecule compounds like Isopropyl 3-(3,4-dihydroxyphenyl)-2-hydroxypropanoate—one of the primary bioactive metabolites of the Chinese medicinal herb Danshen—and Citrulline have shown promise in reducing endothelial cell pyroptosis by inhibiting NLRP3 activity (Xue et al., 2022; Zhang ML. et al., 2023). These discoveries pave the way for new avenues in ALI/ARDS therapeutic strategies. Thirdly, from an immunological standpoint, the relationship between the complement system and endothelial cell pyroptosis has been validated. Specifically, complement component C3a, through its C3aR receptor, can effectively reduce endothelial cell pyroptosis, potentially mitigating sepsis-induced ALI (Li et al., 2022). Fourthly, in the context of new signaling pathways and regulatory factors, phospholipid scramblase 4 has been identified in recent studies (Liu X. et al., 2021) as having a potential regulatory role, which holds significant implications for further elucidating the complex mechanisms of endothelial cell pyroptosis. Taking all these studies into account, the multifaceted roles and diverse regulatory factors involved in endothelial cell pyroptosis in the pathogenesis of ALI/ARDS are gradually being uncovered. However, these studies also introduce new challenges and questions, such as the safety and efficacy of newly discovered signaling pathways or regulatory factors, and possible interactions and cross-regulations between different pathways, all of which necessitate further extensive and in-depth research.

Through keyword co-occurrence network analysis, we found that endothelial cell pyroptosis resides in a diversified keyword cluster in ALI/ARDS research. This cluster includes not only endothelial cell pyroptosis itself but also “TLR/TLR2/TLR4”, “HMGB1”, “Caspase-1/Caspase-3/Caspase-11″, “heparin”, “Necroptosis”, and “HIF/HIF1 alpha”, among others. Firstly, HMGB1 and Caspase-1, which were previously discussed, are closely associated with endothelial cell pyroptosis. Secondly, TLRs are considered upstream signals involved in activating NLRP3 inflammasomes and are explicitly related to endothelial cell pyroptosis (Kinra et al., 2022; Lyu et al., 2023). Notably, heparin, a widely used anticoagulant, has been studied for its role in sepsis-induced ALI, where it can reduce sepsis-related mortality and alleviate ALI by inhibiting Caspase-11-dependent inflammatory responses (Yang et al., 2016). However, the regulatory mechanisms through which heparin impacts lung endothelial cell pyroptosis remain unclear, offering new directions for subsequent research. Additionally, although “HIF/HIF1 alpha” has been investigated in the ALI/ARDS field, its direct association with endothelial cell pyroptosis has yet to be reported, opening new possibilities for future studies. Overall, this keyword cluster underscores the complexity and diversity of ALI/ARDS and endothelial cell pyroptosis research, revealing multiple potential directions for future investigations.

#### 4.2.4 Toll-like receptors

“TLR/TLR2/TLR4” are all members of the Toll-like Receptors (TLRs) family. TLRs serve as essential pattern recognition receptors (PRRs) in the innate immune system, capable of recognizing and binding both pathogen-associated molecular patterns (PAMPs) and damage-associated molecular patterns (DAMPs) (Fitzgerald and Kagan, 2020). Activation of TLRs predominantly triggers downstream signaling pathways such as NF-κB, subsequently leading to the expression of inflammatory and chemotactic cytokines. Recent research in ALI/ARDS has shown that the expression and activity of TLRs in ECs are subject to multifaceted regulation. On the protein level, Rab26 GTPase and USP18 have been identified to significantly modulate the TLR4 and its downstream NF-κB/ROS signaling pathways, making them potential therapeutic targets for inflammation and lung injury (Chen et al., 2019; Jiang et al., 2021). Non-coding RNAs such as miR-146a-5p, lncRNA RP11-490M8.1, and miR-16 negatively regulate TLR4 or TLR7 and their downstream NF-κB pathways, thereby affecting EC functions (Yang et al., 2019; Liu XH. et al., 2021; Huang et al., 2022). Additionally, lncRNA PRNCR1 acts as a competitive endogenous RNA that sequesters miR-330-5p, thereby enhancing TLR4 expression and intensifying the inflammatory response (Yu et al., 2021). In terms of exogenous molecules, extracellular histones H3 and H4 exacerbate acute lung injury through a TLR4-dependent mechanism (Kim et al., 2022; Ramasubramanian et al., 2022), while extracellular nicotinamide phosphoribosyltransferase, acting as a ligand for TLR4, also promotes inflammation and lung injury (Quijada et al., 2021). Moreover, Histone Deacetylase 6 (HDAC6) and Angiopoietin-1 (ANGPT1) play roles in the regulation of TLRs: HDAC6 negatively modulates TLR4 signaling, ameliorating inflammation and lung injury (Menden et al., 2019), whereas ANGPT1 can inhibit LPS-induced pulmonary TLR signaling pathway activation through preconditioning, thereby reducing inflammation and ALI incidence (Salimi et al., 2022). Collectively, these studies reveal an intricate regulatory network of TLRs in ECs, involving a multitude of proteins, non-coding RNAs, and exogenous molecules. This not only enriches our understanding of the functional mechanisms of TLRs in ALI/ARDS but also offers a series of potential therapeutic targets and strategies.

Among other author keywords that co-occur in the same network cluster as “TLR/TLR2/TLR4”, “pyroptosis” and “caspase-1/caspase-3/caspase-11” have been previously discussed. Subsequently, HMGB1, a widely studied DAMP, primarily targets TLR4. Modulating the HMGB1/TLR-4 pathway can mitigate systemic inflammatory responses and ameliorate histopathological changes and microvascular permeability in a rat model of ALI (Ji et al., 2018; Yang et al., 2020). Keywords such as “HIF/HIF1 alpha”, “heparin”, and “necroptosis” have yet to show significant intersections with TLRs in the realm of ALI/ARDS and EC research, signifying uncharted avenues for future investigations in this field.

#### 4.2.5 Nuclear Factor-kappa B

Nuclear Factor-kappa B (NF-κB) is fundamentally a family of transcription factors involved in regulating inflammation, immune responses, and various other physiological and pathological processes (Yu et al., 2020). Its signaling pathways can be categorized into canonical and noncanonical types, each having unique regulatory mechanisms (Yu et al., 2020). The canonical NF-κB pathway is highly emphasized in ALI/ARDS research concerning ECs, where it plays a pivotal role in acute inflammation and immune responses. Recent studies have identified molecules like Sirtuin3 (Li and Shi, 2020), Salidroside (Zhang H. et al., 2021), and gypenosides (Tu et al., 2021) that attenuate pulmonary EC injury by inhibiting canonical NF-κB pathway activity, reducing the severity of LPS-induced ALI/ARDS. These molecules are therefore considered potential ALI/ARDS therapeutic agents. Emerging molecules like TRIM47 (Qian et al., 2022), Moesin (Chen et al., 2021), and Fibroblast growth factor 21 (Zhou et al., 2022) have also garnered attention. These molecules can influence the activation of NF-κB pathways through various mechanisms, inducing inflammation or other pathological effects in ECs, and are considered new research foci and potential therapeutic targets. Although the noncanonical NF-κB pathway primarily functions in long-term cell survival and lymphoid organ development (Yu et al., 2020), preliminary evidence from two studies suggest its potential role in EC damage in ALI/ARDS (Saxon et al., 2016; Fan et al., 2023).

In the research landscape of ALI/ARDS and ECs, NF-κB interacts closely with multiple key factors including “oxidative stress”, “ICAM-1”, “VEGF/VEGFA”, “STAT3/STAT5”, “HO-1”, and “MPO.” For instance, activation of NF-κB can promote ICAM-1 expression, thereby enhancing leukocyte-endothelial cell adhesion, a critical process in the development of ALI (Li G. et al., 2023). Myeloperoxidase (MPO) is often used as a biomarker for evaluating inflammation and oxidative stress in ALI/ARDS. Although NF-κB and MPO are frequently co-assayed in research related to ALI/ARDS and ECs, direct evidence confirming whether NF-κB explicitly regulates MPO expression or activity is currently lacking (Mao and Wu, 2022). Other key factors like “HO-1”, “VEGF/VEGFA”, and “STAT3/STAT5” are also related to NF-κB in ECs and ALI/ARDS research (Huang et al., 2019; Zhang H. et al., 2023; Chang and Zhang, 2023). Despite these observed interactions in various studies, the intricate mechanisms and interplays remain a focus for future research. Overall, NF-κB exhibits definitive interactions with a multitude of biomarkers and signaling pathways associated with ALI/ARDS and ECs. While these associations have been observed in different studies, the biological significance and mechanistic details of these interactions require further in-depth investigation.

#### 4.2.6 NLR-family pyrin domain-containing protein 3

NLR-family pyrin domain-containing protein 3 (NLRP3) is a crucial inflammasome in the innate immune system. As high molecular weight protein complexes, inflammasomes serve as intracellular receptors in innate immunity, sensing PAMPs or DAMPs and mediating heightened inflammatory states (Zhang WJ. et al., 2023). The NLRP3 inflammasome is among the most comprehensively studied and characterized polyprotein complexes to date (Bai et al., 2020). Given its close association with pyroptosis, NLRP3 often appears in ALI/ARDS and ECs research; its current status has been detailed in the section on pyroptosis. Beyond cellular pyroptosis, activation of the NLRP3 inflammasome also triggers the secretion of mature IL-1β and IL-18, further inducing inflammatory processes and oxidative stress within the endothelium (Bai et al., 2020). Recent research has uncovered the potential therapeutic applications of NLRP3 inflammasomes in ALI/ARDS. A study found that Yam Glycoprotein, extracted from traditional Chinese medicine, significantly mitigated lung tissue damage and inflammation in LPS-induced ALI mouse models (Niu et al., 2020). This study accentuated the pivotal role of NLRP3 inflammasomes within the TLR4/NF-κB signaling pathway, suggesting that interventions targeting this molecular node could ameliorate ALI pathophysiology effectively. Additionally, a Chinese herbal formula named Shiwei Qingwen decoction demonstrated its ability to attenuate inflammation in LPS-induced ALI by inhibiting NLRP3 inflammasomes (Zhang Q. et al., 2023). These findings further substantiate the NLRP3 inflammasome as an essential regulator for both inflammatory modulation and tissue repair. In summary, the role of the NLRP3 inflammasome in ALI/ARDS and ECs research extends beyond cellular pyroptosis to encompass broader inflammatory mechanisms and tissue protection, offering new research avenues and therapeutic possibilities.

Regarding NLRP3 research in ALI/ARDS and ECs, the current volume of literature is limited, with only six papers having NLRP3 as a keyword. Consequently, in keyword clustering analysis, connections between NLRP3 and other research areas are not significantly pronounced. One notable intersection is the relationship between SIRT1 and NLRP3. Existing research indicates that upregulation of SIRT1 effectively inhibits NLRP3 inflammasome activation, thereby attenuating EC pyroptosis and the progression of lung inflammation. This places SIRT1 as a potential new therapeutic target for inhibiting NLRP3 inflammasome activation in the treatment of ALI/ARDS and ECs. As for other keywords like “endothelial glycocalyx”, “miRNA”, “angiogenesis”, “endothelial progenitor cells”, “extracellular vesicles (EVs)", “exosomes”, “heparanase”, and “Focal adhesion kinase (FAK)”, no literature currently examines their roles with NLRP3 in ALI/ARDS and ECs, pointing to future research directions.

In summary, this study systematically analyzes the intricate interplay between ECs and ALI/ARDS, identifying six author keywords as focal points and frontiers in current research. Specifically, the paper elaborates on the significance of endothelial glycocalyx, oxidative stress, pyroptosis, TLRs, NF-κB, and NLRP3 in the investigation of ECs and ALI/ARDS. These keywords encompass a range of topics, from the external structure of ECs to redox reactions, and on to specific cellular death mechanisms and intracellular signaling molecules or pathways within the immune system, showcasing the multifaceted role of ECs in the pathophysiology of ALI/ARDS. Each keyword not only delineates the current state of research but also highlights directions warranting further exploration.

### 4.3 Strengths and limitations

Strengths: First, this study is the inaugural comprehensive evaluation of the development trends in the ALI/ARDS and ECs research field using bibliometric methods, providing scholars with a systematic reference. Second, the Python code utilized in this study is capable of automatically reading txt files containing citation information from articles, swiftly generating bubble charts to display the research findings. Lastly, the code is original work by the author and has been open-sourced on the GitHub platform, making it convenient for other researchers to modify or extend it according to their needs.

Limitations: First, although the WoSCC database relied upon for this study is widely considered an authoritative resource in the realm of scientific publishing, it does not include articles from non-SCI journals or other databases, potentially leading to the omission of some studies. Second, since bibliometric analysis primarily depends on citation metrics, the methodology cannot comprehensively evaluate the intrinsic quality of individual articles. Additionally, citation frequency is affected by the temporal factor, often resulting in fewer citations for more recent studies. Lastly, articles lacking author-defined keywords were not included in this analysis, a limitation that may exert some influence on the analytical outcomes. Despite these limitations, they are unlikely to alter the primary trends revealed by this study.

## 5 Conclusion

Employing bibliometric methodologies in conjunction with Python programming language, VOSviewer, and Citespace software, this study offers a comprehensive analysis of ALI/ARDS and ECs research articles published in WoSCC database from 2011 to 2023. The findings indicate that this field has consistently remained a focal point in scientific research since 2011. Globally, the United States leads in the volume of publications, research institutional impact, and the number of top-tier scholars, followed by Germany and China. Through an in-depth examination of author keywords, this study identifies endothelial glycocalyx, oxidative stress, pyroptosis, TLRs, NF-κB, and NLRP3 as key research hotspots and potential frontier directions in this field. These subjects not only elucidate the intricate interactions between ALI/ARDS and ECs but also point to prospective avenues for future research. This study may aid newcomers in rapidly and clearly understanding the current global landscape of ALI/ARDS and ECs research and offers valuable reference information for institutions or groups seeking collaborative research opportunities.

## Data Availability

The datasets presented in this study can be found in online repositories. The names of the repository/repositories and accession number(s) can be found below: https://github.com/changecool/WOS_bibliometric-analysis.
