# Supplementary material for: Global research progress of endothelial cells and ALI/ARDS: a bibliometric analysis
Source: Front Physiol. 2024 May 7;15:1326392. doi: 10.3389/fphys.2024.1326392 (PMC11107300; doi:10.3389/fphys.2024.1326392)
Supplement: Supplementary file 1 [file Table1.DOCX]

Supplementary Material

**Supplementary Table 1.** Contribution of the top 20 research areas in ALI/ARDS and ECs.

| Rank | WOS research area | TP | TC | h-index | ACPP | TPR (%) |
| --- | --- | --- | --- | --- | --- | --- |
| 1 | Cell Biology | 199 | 4691 | 38 | 23.57 | 20.47 |
| 2 | Respiratory System | 182 | 6885 | 46 | 37.83 | 18.72 |
| 3 | Biochemistry & Molecular Biology | 164 | 3277 | 33 | 19.98 | 16.87 |
| 4 | Medicine, Research & Experimental | 122 | 3008 | 28 | 24.66 | 12.55 |
| 5 | Physiology | 115 | 3036 | 33 | 26.4 | 11.83 |
| 6 | Pharmacology & Pharmacy | 114 | 1741 | 25 | 15.27 | 11.73 |
| 7 | Immunology | 111 | 2576 | 28 | 23.21 | 11.42 |
| 8 | Hematology | 64 | 1793 | 24 | 28.02 | 6.58 |
| 9 | Multidisciplinary Sciences | 57 | 2651 | 27 | 46.51 | 5.86 |
| 10 | Critical Care Medicine | 55 | 2093 | 25 | 38.05 | 5.66 |
| 11 | Peripheral Vascular Disease | 53 | 1511 | 22 | 28.51 | 5.45 |
| 12 | Surgery | 43 | 773 | 19 | 17.98 | 4.42 |
| 13 | Oncology | 31 | 641 | 13 | 20.68 | 3.19 |
| 14 | Cardiac & Cardiovascular Systems | 28 | 680 | 12 | 24.29 | 2.88 |
| 15 | Pathology | 26 | 802 | 12 | 30.85 | 2.67 |
| 16 | Medicine, General & Internal | 25 | 372 | 11 | 14.88 | 2.57 |
| 17 | Biology | 24 | 542 | 12 | 22.58 | 2.47 |
| 18 | Chemistry, Multidisciplinary | 22 | 466 | 10 | 21.18 | 2.26 |
| 19 | Toxicology | 21 | 243 | 10 | 11.57 | 2.16 |
| 20 | Biophysics | 18 | 232 | 10 | 12.89 | 1.85 |

TP, total papers; TC, total citations; ACPP, average citations per publication; and TPR, the percentage of articles of institutions in total publications.

**Supplementary Table 2.** All highly cited ESI publications in ALI/ARDS and ECs from 2011 to 2023.

| Rank | authors | Title | Type | TC | Publication year | Journal |
| --- | --- | --- | --- | --- | --- | --- |
| 1 | Abrams, Simon T. et al. | Circulating Histones Are Mediators of Trauma-associated Lung Injury | Article | 378 | 2013 | American Journal of Respiratory and Critical Care Medicine |
| 2 | Hojyo, Shintaro et al. | How COVID-19 induces cytokine storm with high mortality | Review | 356 | 2020 | Inflammation and Regeneration |
| 3 | Short, Kirsty R. et al. | Pathogenesis of influenza-induced acute respiratory distress syndrome | Review | 334 | 2014 | Lancet Infectious Diseases |
| 4 | Herold, Susanne et al. | Influenza virus-induced lung injury: pathogenesis and implications for treatment | Article | 262 | 2015 | European Respiratory Journal |
| 5 | Schaefer, Inga-Marie et al. | In situ detection of SARS-CoV-2 in lungs and airways of patients with COVID-19 | Article | 190 | 2020 | Modern Pathology |
| 6 | van de Veerdonk, Frank L. et al. | Kallikrein-kinin blockade in patients with COVID-19 to prevent acute respiratory distress syndrome | Article | 189 | 2020 | eLife |
| 7 | Matthay, Michael A. et al. | Clinical trials in acute respiratory distress syndrome: challenges and opportunities | Review | 180 | 2017 | Lancet Respiratory Medicine |
| 8 | Whyte, Claire S. et al. | Fibrinolytic abnormalities in acute respiratory distress syndrome (ARDS) and versatility of thrombolytic drugs to treat COVID-19 | Review | 175 | 2020 | Journal of Thrombosis and Haemostasis |
| 9 | Lamers, Mart M. et al. | SARS-CoV-2 pathogenesis | Review | 161 | 2022 | Nature Reviews Microbiology |
| 10 | Kang, Sujin et al. | IL-6 trans-signaling induces plasminogen activator inhibitor-1 from vascular endothelial cells in cytokine release syndrome | Article | 156 | 2020 | Proceedings of the National Academy of Sciences of the United States of America |
| 11 | Batah, Sabrina Setembre et al. | Pulmonary pathology of ARDS in COVID-19: A pathological review for clinicians | Review | 140 | 2021 | Respiratory Medicine |
| 12 | Acosta, Manuel A. Torres et al. | Pathogenesis of COVID-19-induced ARDS: implications for an ageing population | Article | 121 | 2020 | European Respiratory Journal |
| 13 | Smadja, David M. et al. | COVID-19 is a systemic vascular hemopathy: insight for mechanistic and clinical aspects | Review | 75 | 2021 | Angiogenesis |
| 14 | Biancatelli, Ruben M. L. Colunga et al. | The SARS-CoV-2 spike protein subunit S1 induces COVID-19-like acute lung injury in K18-hACE2 transgenic mice and barrier dysfunction in human endothelial cells | Article | 60 | 2021 | American Journal of Physiology-Lung Cellular and Molecular Physiology |
| 15 | Silva, Johnatas Dutra et al. | Mesenchymal stromal cell extracellular vesicles rescue mitochondrial dysfunction and improve barrier integrity in clinically relevant models of ARDS | Article | 56 | 2021 | European Respiratory Journal |
| 16 | Otifi, Hassan M. et al. | Endothelial Dysfunction in Covid-19 Infection | Review | 36 | 2022 | American Journal of the Medical Sciences |

TC, total citations.
